# Possibilities and limitations of intravital imaging

**DOI:** 10.17179/excli2016-863

**Published:** 2016-12-23

**Authors:** Reham Hassan

**Affiliations:** 1 Forensic Medicine and Toxicology Department, Faculty of Veterinary Medicine, South Valley University, Qena, Egypt

## ⁯⁯

Recently, Raymond Reif and colleagues from the Leibniz Research Center in Dortmund have published a technical article about two-photon based intravital imaging (Reif et al., 2016[16]). The strength of this article is the outstanding quality of the presented videos, which show organs of living mice, namely liver, kidney and intestine, with a resolution of approximately 200 nm. Key features of the established imaging technique are two-photon lasers with a broad wavelength spectrum, long-distance objectives with high numerical aperture, a sensitive detector and an optimized anesthesia (Reif et al., 2016[16]). Under these conditions the technique can be applied to multiple organs to study transport and elimination of xenobiotics and endogenous compounds. A fundamental prerequisite of intravital imaging is the use of appropriate fluorophore-coupled compounds and fluorescent reporter mice. The authors used this method to image and quantify the flux of fluorescent bile salts and drugs from the sinusoids of the liver through the Dissé space into hepatocytes and finally into bile canaliculi. In the kidney it was possible to analyze glomerular filtration into the Bowman´s capsule followed by transport into the proximal and then to the distal tubules. After bolus injections of fluorescent compounds even the passage time from proximal to distal tubules via the Henle loop could be determined. Finally, the same approach was used to image intestinal crypts and study the transfer of drugs from capillaries to lymph vessels.

The presented intravital technique has major implications for three fields of research. First, pharmacokinetic modelling can be improved by intravital imaging. PBPK modelling is of high relevance for simulation of compound concentrations in blood or organs (Golubovskaya et al., 2015[9]; Stamyr et al., 2015[20]; Widera, 2015[24]; Ghallab, 2015[5]) as well as for interspecies extrapolation (Thiel et al., 2015[21]). However, current PBPK models consider individual organs as single compartments (Schug et al., 2013[19]; Mielke et al., 2011[14]). Using intravital microscopy it became clear that sub-compartments of tissues may accumulate specific compounds. Integrating these dynamics into PBPK models may further improve simulations. Second, establishment of in vitro systems represents a cutting-edge topic (Ghallab and Bolt, 2014[6]; Godoy et al., 2013[8]; Krug et al., 2013[13]), particularly in the fields of cell systems for hepatotoxicity (Reif et al., 2015[17]; Böttger et al., 2015[1]; Grinberg et al., 2014[10]), nephrotoxicity (Wassermann et al., 2013[23]; Valente et al., 2012[22]; Buhrke et al., 2015[2]) and intestinal transport (Niu et al., 2015[15]). It is important to be aware of similarities but also differences of these cultivated cells to their in vivo counterparts. Two-photon imaging can be used to study compound transport in vitro and in vivo by precisely the same method. Third, spatio-temporal modelling has become a well-established technique in systems biology (Drasdo et al., 2014[3][4]; Ghallab et al., 2016[7]; Hammad et al., 2014[11]). A basic principle of spatio-temporal modelling is that tissues are reconstructed in a way that the position of each cell in a three-dimensional space is known; next further processes of interest, e.g. metabolic models, can be programmed into each individual cell to simulate the consequences at the tissue level (Hoehme et al., 2010[12]; Schliess et al., 2014[18]). Two-photon based imaging represents an excellent technique to validate predictions from spatio-temporal tissue models.

In conclusion, the two-photon based imaging technique presented by Reif and colleagues will support spatio-temporal modelling studies of physiological and pathophysiological processes in all organs of interest. 
